# Supplementary material for: The impact of physical activity on subjective well-being: The mediating role of exercise identity and the moderating role of health consciousness
Source: PLoS One. 2025 Apr 23;20(4):e0313799. doi: 10.1371/journal.pone.0313799 (PMC12017510; doi:10.1371/journal.pone.0313799)
Supplement: Attachment — Submitted filename: Response to Reviewers.docx [file pone.0313799.s003.docx]

**Response to Reviewers**

We sincerely thank the editor and the reviewer for their comments and advice, which we have used to improve the manuscript. Point-by-point responses to each comment follow below. The line numbers refer to the revised manuscript.

**Journal Requirements:**

**Comment 1:** Please ensure that your manuscript meets PLOS ONE's style requirements, including those for file naming. The PLOS ONE style templates can be found at [*https://journals.plos.org/plosone/s/file?id=wjVg/PLOSOne_formatting_sample_main_body.pdf*](https://journals.plos.org/plosone/s/file?id=wjVg/PLOSOne_formatting_sample_main_body.pdf) and [*https://journals.plos.org/plosone/s/file?id=ba62/PLOSOne_formatting_sample_title_authors_affiliations.pdf*](https://journals.plos.org/plosone/s/file?id=ba62/PLOSOne_formatting_sample_title_authors_affiliations.pdf).

**Response:**
Thank you for your comment. We have carefully reviewed the PLOS ONE style requirements and revised our manuscript to ensure full compliance with the guidelines. The file naming conventions have also been updated accordingly. Additionally, we have consulted the style templates provided at the links and formatted the manuscript to align with these templates.

**Comment 2:** *Please include your full ethics statement in the ‘Methods’ section of your manuscript file. In your statement, please include the full name of the IRB or ethics committee who approved or waived your study, as well as whether or not you obtained informed written or verbal consent. If consent was waived for your study, please include this information in your statement as well.*

**Response:**
Thank you for your valuable feedback. We have updated the ‘Methods’ section of our manuscript to include the full ethics statement. The statement now specifies the name of the Institutional Review Board (IRB) that approved the study—Guangzhou Sports University Ethics Committee. It also clarifies that informed written consent was obtained from all participants (p. 6, 126–130).

**Comment 3:** *Please include captions for your Supporting Information files at the end of your manuscript, and update any in-text citations to match accordingly. Please see our Supporting Information guidelines for more information:* [*http://journals.plos.org/plosone/s/supporting-information*](http://journals.plos.org/plosone/s/supporting-information)*.*

**Response:**
Thank you for your comment. We have revised the manuscript according to the reviewers' comments as requested.

**Comment 4:** We are unable to open your Supporting Information file “data.sav.” Please kindly revise as necessary and re-upload.

**Response:**
Thank you for bringing this issue to our attention. Upon review, we have converted the file to Excel format.

**Response to Reviewer**

**Comment 1:** *Is the manuscript presented in an intelligible fashion and written in standard English? PLOS ONE does not copyedit accepted manuscripts, so the language in submitted articles must be clear, correct, and unambiguous. Any typographical or grammatical errors should be corrected at revision, so please note any specific errors here. Reviewer #1: No*

**Response:**
Thank you for your feedback. The manuscript was edited for proper English language, grammar, punctuation, spelling, and overall style by one or more of the highly qualified English speaking editors at SNAS. All typographical and grammatical errors identified have been corrected. Additionally, we have conducted a thorough review of the language to ensure that it adheres to the standards of academic English.

**Comment 2:** *The basic characteristics of the subjects are described too little, such as occupation, family or personal income, and marital status. This information needs to be supplemented.*

**Response:**
Thank you for your valuable comment. In the revised manuscript, we have supplemented the description of the subjects’ basic characteristics by including information on their occupation, family or personal income, and marital status. This additional information is now detailed in the ‘Participants’ subsection of the Methods section (p. 6, 117–125).

**Comment 3:** *The hypothesis of Figure 1 is flawed. Why is there a modulatory effect only assumed for the mediating path, but not for the direct path? A schematic representation of the moderating effect of only one pathway is drawn, and it should be assumed that health awareness has a moderating effect on all three pathways.*

**Response:**
Thank you for your insightful comment regarding the hypotheses in Figure 1. We agree that it is important to consider the potential moderating effects of health awareness comprehensively. In the revised manuscript, we have supplemented the theoretical rationale to justify why the moderating effect of health awareness was initially hypothesized only for the mediating path (p. 5, 85-101).

**Comment 4:** Health identity has a complete mediating effect, and health awareness should moderate the two mediating pathways.

**Response:** Thank you for your suggestion. We have revised the manuscript accordingly (p. 5, 85-101).

**Comment 5:** What is the difference between MODEL 1 and MODEL 4 in Table 1? Model 1 has 4 factors, right? Model 4 only has one factor? It needs to be explained further.

**Response:**
Thank you for pointing out the need for further clarification regarding the differences between MODEL 1 and MODEL 4 in Table 1. We have addressed this issue in the revised manuscript. Specifically, we added a detailed explanation of the differences between these models, including their respective factors and theoretical implications (p. 9, 180-184).

**Comment 6:** *The three variables of SEX, AGE, and EDUCATION in Table 2 should be used as adjusted confounding variables, rather than as the main analysis variables. It is better to do partial correlation analysis with adjusted confounding variables.*

**Response:**
Thank you for this valuable comment. We have carefully revised the manuscript to address your concerns. Specifically, we have reanalyzed the data to treat SEX, AGE, and EDUCATION as adjusted confounding variables rather than as the main analysis variables. Partial correlation analyses have been conducted accordingly, and the results have been updated in Table 2 to reflect this adjustment (p. 9).

**Comment 7:** *In Table 2, the correlation between physical activity, health awareness, and health identity is close to 0.6, and the variables are almost interchangeable. Is there a problem in the selection of the scale or the measurement process?*

**Response:**
Thank you for raising this important concern. To address it, we conducted a thorough review of the scales used and the measurement process to ensure their appropriateness. We confirmed that the scales for physical activity, health awareness, and health identity were selected on the basis of established theoretical frameworks and validated in prior studies.

Additionally, we have included a detailed explanation in the manuscript to clarify the rationale for scale selection, as well as the steps taken to minimize measurement overlap. (pp. 9-10, 190-210)

**Comment 8:** *Neither Figure 1 nor Figure 2 is clearly expressed.*

**Response:**
Thank you for your valuable feedback. We have carefully reviewed Figures 1 and 2 to address the clarity issues you raised.

1. **Figure 1:** We have provided additional explanations for Table 1 in the manuscript to clarify the relationships and variables depicted in Figure 1. These modifications aim to ensure that the figure aligns more closely with the theoretical framework and is easier to interpret.
2. **Figure 2:** We have redrawn Figure 2 to improve its clarity and visual representation. The updated figure uses a more structured layout, clearer labeling, and consistent formatting to increase readability.

**Comment 9:** 62.9% are bachelor degree and 15.6% are master degree. The study population was highly educated, and the extrapolation of the findings was severely limited and not mentioned in the Limitation.

**Response:**
Thank you for pointing out this important limitation of our study. We have addressed your concern by adding a detailed explanation of the limitation regarding the high educational level of the study population. This discussion highlights how the findings might not be fully generalizable to populations with different educational backgrounds (p. 18, 345-352).
