# Supplementary material for: The impact of physical activity on subjective well-being: The mediating role of exercise identity and the moderating role of health consciousness
Source: PLoS One. 2025 Apr 23;20(4):e0313799. doi: 10.1371/journal.pone.0313799 (PMC12017510; doi:10.1371/journal.pone.0313799)
Supplement: Attachment — Submitted filename: Response to Reviewers_0206.docx [file pone.0313799.s004.docx]

**Response to Reviewers**

We sincerely thank the editor and the reviewer for their comments and advice, which we have used to improve the manuscript. Point-by-point responses to each comment follow below. The line numbers refer to the revised manuscript.

**Journal Requirements:**

**Comment 1:** *Please review your reference list to ensure that it is complete and correct. If you have cited papers that have been retracted, please include the rationale for doing so in the manuscript text, or remove these references and replace them with relevant current references. Any changes to the reference list should be mentioned in the rebuttal letter that accompanies your revised manuscript. If you need to cite a retracted article, indicate the article’s retracted status in the References list and also include a citation and full reference for the retraction notice.*

**Response:**
We have carefully reviewed all references to ensure their completeness and accuracy. No retracted papers were cited in our manuscript. Therefore, no changes were necessary. If further adjustments are required, we are happy to make them.

**Response to Reviewer 2**

**Comment 1:** *Method: In the measures section, the name of the scale used can be listed as a single line of subheadings. Common methodological biases can be placed in the results section. The content of reliability and validity analysis can be separately placed in the introduction of relevant scales in the measures.*

**Response:**

We sincerely appreciate the reviewer’s valuable suggestions regarding the structure of the Measures section. In response, we have made the following revisions:

1. We have listed the names of the scales as single-line subheadings to improve clarity and readability (p. 7, 114, 144, 148, 152).
2. Common methodological biases have been moved to the Results section, as suggested (p. 9, 181–186).
3. The reliability and validity analyses remain in the Methods section but have been placed after the introduction of the scales to ensure a logical flow and better distinction between the measurement tools and their psychometric properties (p. 8, 158).

These changes enhance the overall structure and clarity of the manuscript while maintaining coherence in presenting the methodological framework. We appreciate the reviewer’s constructive feedback and the opportunity to improve our work.

**Comment 2:** *Conclusion and discussion: The writing format of this part does not conform to the standard of writing academic papers. Usually write the discussion first and the conclusion last. In addition, the content of the conclusion mainly describes the content of the research results, rather than the research conclusion.*

**Response:**
Thank you for your valuable feedback regarding the structure of the Discussion and Conclusion sections. In response to your comment, I have made the following adjustments:

1. I have restructured the manuscript to place the Discussion section before the Conclusion, in accordance with academic writing standards (p. 14, 249, 250, 287, 331).
2. I have revised the Conclusion to focus on summarizing the research findings rather than reiterating the content of the study. The revised conclusion now presents the key results and insights derived from the study, offering a clear and concise summary (p. 19, 353–362).
